# Understanding genetic diversity in drought-adaptive hybrid parental lines in pearl millet

**DOI:** 10.1371/journal.pone.0298636

**Published:** 2024-02-23

**Authors:** Kuldeep Kandarkar, Viswanathan Palaniappan, Subhrajit Satpathy, Anilkumar Vemula, Ravikesavan Rajasekaran, Prabhakaran Jeyakumar, Nakkeeran Sevugaperumal, Shashi Kumar Gupta

**Affiliations:** 1 Centre for Plant Breeding and Genetics, Tamil Nadu Agricultural University, Coimbatore, Tamil Nadu, India; 2 International Crops Research Institute for the Semi-Arid Tropics (ICRISAT), Patancheru, Hyderabad, Telangana, India; 3 Department of Crop Physiology, Tamil Nadu Agricultural University, Coimbatore, Tamil Nadu, India; 4 Department of Plant Pathology, Tamil Nadu Agricultural University, Coimbatore, Tamil Nadu, India; KGUT: Graduate University of Advanced Technology, ISLAMIC REPUBLIC OF IRAN

## Abstract

Information on genetic diversity and population structure is helpful to strategize enhancing the genetic base of hybrid parental lines in breeding programs. The present study determined the population structure and genetic diversity of 109 pearl millet hybrid parental lines, known for their better adaptation and performance in drought-prone environments, using 16,472 single nucleotide polymorphic (SNP) markers generated from GBS (genotyping-by-sequencing) platforms. The SNPs were distributed uniformly across the pearl millet genome and showed considerable genetic diversity (0.337), expected heterozygosity (0.334), and observed heterozygosity (0.031). Most of the pairs of lines (78.36%) had Identity-by-State (IBS) based genetic distances of more than 0.3, indicating a significant amount of genetic diversity among the parental lines. Bayesian model-based population stratification, neighbor-joining phylogenetic analysis, and principal coordinate analysis (PCoA) differentiated all hybrid parental lines into two clear-cut major groups, one each for seed parents (B-lines) and pollinators (R-lines). Majority of parental lines sharing common parentages were found grouped in the same cluster. Analysis of molecular variance (AMOVA) revealed 7% of the variation among subpopulations, and 93% of the variation was attributable to within sub-populations. Chromosome 3 had the highest number of LD regions. Genomic LD decay distance was 0.69 Mb and varied across the different chromosomes. Genetic diversity based on 11 agro-morphological and grain quality traits also suggested that the majority of the B- and R-lines were grouped into two major clusters with few overlaps. In addition, the combined analysis of phenotypic and genotypic data showed similarities in the population grouping patterns. The present study revealed the uniqueness of most of the inbred lines, which can be a valuable source of new alleles and help breeders to utilize these inbred lines for the development of hybrids in drought-prone environments.

## Introduction

Pearl millet (*Pennisetum glaucum* (L.) R. Br.) is the sixth most important cereal grown on about 30 mha in arid and semi-arid areas across the globe. It accounts for more than 50 percent of the worldwide production of millets [1]. It is a highly cross-pollinated C4 panicoid grass with an excellent photosynthetic efficiency and biomass production. Pearl millet is a short annual diploid crop (2n = 2x = 14) with a draft assembly genome size of ~ 1.79 Gb [2] It is a staple crop for more than 500 million people and an integral part of the agro-ecosystem of marginal farmers from arid and semi-arid regions of sub-Saharan Africa, India, and South Asia.

India is the largest producer of pearl millet with 7.6 mha and production of 10.86 mt with average productivity of 1420 kg/ha [3]. Approximately 70 percent of pearl millet cultivations in India are hybrids. The larger areas of north-western India, comprising areas of Rajasthan, Gujarat, and Haryana states, are drought-prone as these areas receive an annual rainfall of less than 400 mm [4]. This ecology occupying almost 3.5 million ha under pearl millet cultivation, is characterized by low and erratic rainfall with poor soils, which leads to very low productivity levels (600–700 kg/ha) under such harsh conditions. The low cultivar diversity in this drought-prone environment exacerbates yield-limiting factors for pearl millet under such challenging conditions. Therefore, the development of climate-smart genotypes and hybrids that are resilient to such conditions can be developed through careful, integrated, and target-trait profile-based well-crafted breeding strategies. Different trait requirements such as earliness, high tillering, high panicle-harvest index, small to medium grain size, optimal biomass (dry fodder) production, and shorter grain filling periods, need to be considered while breeding for this drought-prone ecology [5]. With genetic diversification of hybrid parents, a higher level of heterosis can be achieved via hybrid breeding in this zone.

Several studies have been conducted regarding molecular marker-based diversity profiling and phenotypic characterization to understand genetic variability and the relationship between the parental genotypes of pearl millet hybrids and/or germplasm [6]. However, most previous studies were based on materials bred for better-endowed ecologies (400–600 mm rainfall/annum). Thus, the present study assessed the phenotypic and genotypic diversity of an existing set of hybrid parental lines known for their adaptation to drought-prone conditions.

## Materials and methods

### Plant material

The current study utilized 109 parental lines, comprising 41 seed parental lines (A/B-lines) and 68 pollinators (R-lines), bred at ICRISAT, Patancheru (S1 Table). The parents were advanced breeding lines (>F5/F6) known for their better performance and adaptation to drought-prone environments. Seed parents were coded as A1/B1 to A41/B41, and pollinators as R1 to R69 (S1 Table). *Tift* 23D2B1, a maintainer of the A1 CMS system, bred at Tifton (USA) [7], was used as the reference genotype.

### DNA extraction and library preparation

For each genotype, almost 15–20 seeds were sown in small pots along with *Tift* 23D2B1 in a glass house at ICRISAT, Patancheru. Leaf tissues were collected from 12–15 days old seedlings, with 5–6 seedlings per accession, each contributing approximately 100 mg of bulk leaf tissue, and stored immediately in a 96-well plate. DNA was isolated using the NucleoSpin® 96 plant II kit (Machey- Nagel, Germany), and elution of DNA was generated for library preparation. To assess the quality of genomic DNA gel electrophoresis (0.8% agarose) was performed in Tri-acetate EDTA buffer in a tank at 100 volts for 60 mins. Qualitative and quantitative checks were performed using a NanoDrop 8000 spectrophotometer, followed by the normalization of genomic DNA (10 ng/μL) was done for further GBS library preparation.

The GBS (Genotyping-By-Sequencing) method was adopted to identify genome-wide SNPs in the hybrid parental lines, as described previously [8]. First, the extracted genomic DNA was digested using ApeKI endonuclease for 2 hr at 75°C and then ligated with adapters with a unique multiplex sequence index (barcode). Next, aliquots of ligated DNA from all samples were pooled and purified to remove excess adapters. The indexed library was purified and analyzed using the Agilent Bioanalyzer 2100 (Agilent, Santa Clara, CA, USA). Subsequently, amplicons were pooled and subjected to PCR amplification. A total of 12 pM from each equimolarly pooled, index-tagged library was loaded onto eight lanes of a high-output v3 flow cell (Illumina p/n PE-401-3001-FC). The cBot-automated cluster generation system (Illumina, SY-301-20020) was employed for single-end cluster generation using the TruSeq SR Cluster Kit v3-cBot-HS (Illumina, GD-401-3001). Sequencing using single-end reads (1 × 101 bp) was carried out at Hiseq2500 platform (Illumina, SY-401-2501) and using TruSeq SBS Kit v3-HS (100 cycles) (Illumina, FC-401-3001). From HiSeq sequencing, we have obtained a total of 294,778,209 raw reads, corresponding to 1,762,042 to 5,451,371 reads per sample (average 2,704,387).

### SNP calling and filtering

**Fig 1:**
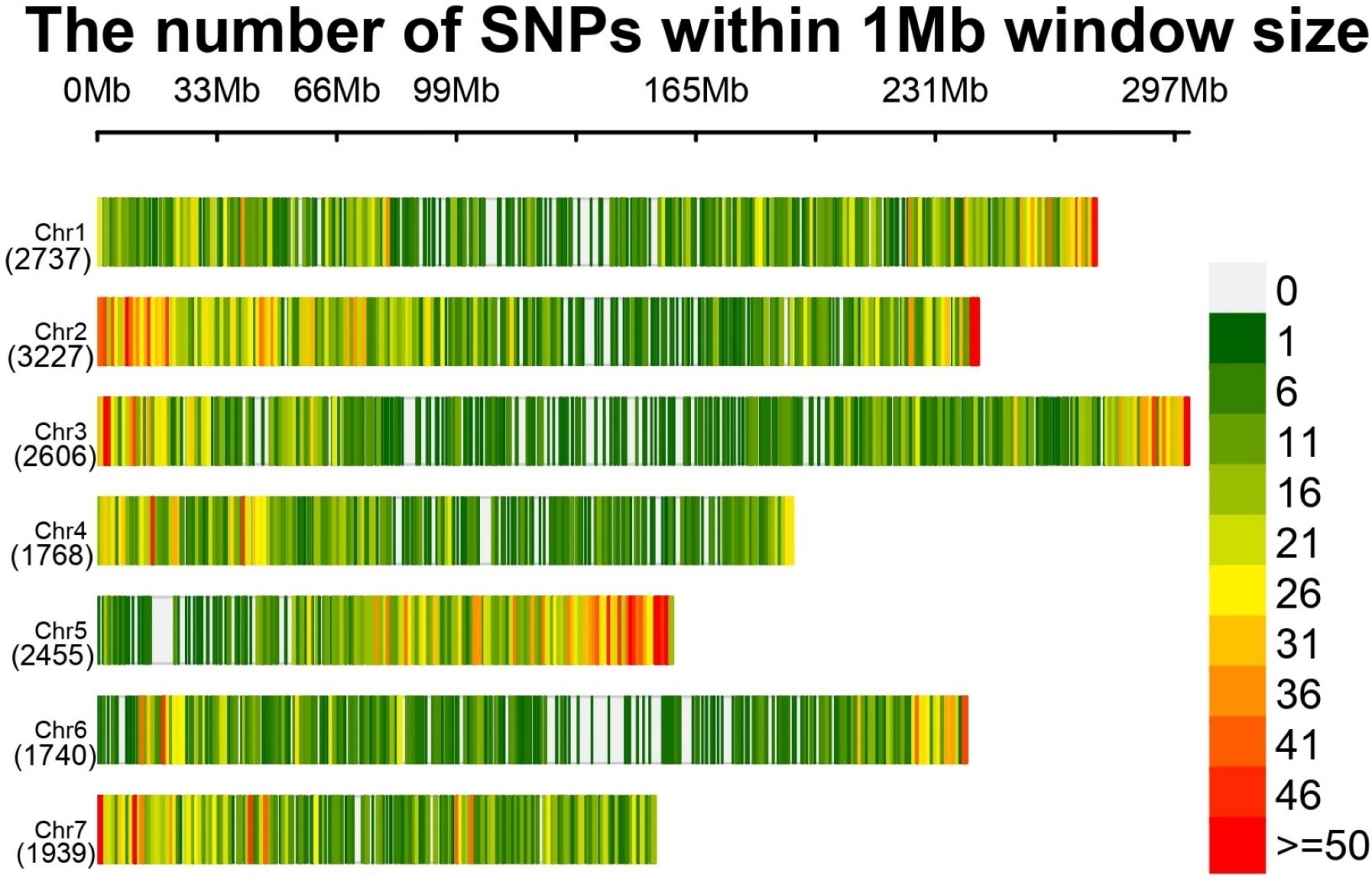
The distribution of genome-wide single-nucleotide polymorphisms (SNPs) of 16,472 SNP markers across the seven chromosomes of pearl millet was identified through Genotyping by Sequencing (GBS) of 109 hybrid parental lines. The number of SNP markers identified for each chromosome is presented in parentheses.

The raw sequence reads obtained from the Illumina platform were aligned to the pearl millet reference genome V1.1 [2] using the default settings of GSNAP [9]. After alignment, the SNPs were called using the software package 123 SNP [10]. The criteria and methodology were adopted to obtain polymorphic SNPs calls as described previously [11]. A total of 7,22,672 raw SNPs were filtered to remove redundant markers with criteria where markers with missing rates of more than 20%, minor allele frequency (MAF) below 5%, and more than 10% of heterozygous calls. Finally, we retained 16,472 SNPs within chromosomes ranging from 1740 (Chr 6) to 3227 (Chr 2), with an average of 2353 SNPs per chromosome (Fig 1). After this, filtered SNP data were subjected to imputation to obtain missing site information using Beagle (Version; 05-05-2022.23a.). To verify the quality of retained SNP markers, we applied earlier defined filtration criteria using VCFtools (Linux-based) [12] and the ’plinkQC’ package in R [13,14]. This ensured the identification of high-quality SNPs. The filtration process was consistent across both tools (the same SNP markers were filtered), reaffirming the reliability of the selected SNPs. Before any quantitative genetic analysis, SNPs or markers with low MAF (less than 5%) were removed [15].

### Population structure, cluster analysis and principal coordinate analysis (PCoA)

For the analysis of population structure, a set of 16,472 single nucleotide polymorphisms (SNPs) distributed across the entire genome was used to assess familial and genetic relatedness. The analysis was performed using a Bayesian model-based program implemented in the STRUCTURE v2.3.4 software package, as recommended by [16]. To explore the population structure, an analysis was conducted using k-values (representing the assumed number of subpopulations) ranging from 1 to 10, with the burn-in length and MCMC cycle set to 100,000. For each k-value, five independent analyses were conducted using an admixture model with correlated allele frequencies. The best k-value could not be readily determined by considering the likelihood value [LnP(D)] of the structure software; hence, the *ad-hoc* delta K [17] was determined to reveal the number of subgroups by implementing the structure output in a structure harvester program (http://taylor0.biology.ucla.edu/structureHarvester/index.php). The population matrix Q for individual accessions was obtained from generated CLUMPP files, and accessions with Q values > 0.6 were in the same group.

Genetic diversity matrix (Identity-By-State based), nucleotide diversity (π), and Tajima’s D statistics for the parental population were estimated using 16,472 SNPs through TASSEL v5.2.82 (Trait Analysis by Association Evolution and Linkage) [18] to further assess the genetic relationship between parental lines. The population grouping pattern obtained from STRUCTURE was further supported by neighbor-joining (NJ) trees based on the Nei’s genetic distance, which was calculated using BIO-R software [19], and NJ trees were constructed using DARwin v6 software [20]. Finally, principal coordinate analysis (PCoA) was performed based on the genetic distance matrix of the parental lines and calculated using GenALEx version 6.5 Software [21].

### Analysis of molecular variance (AMOVA) and genetic diversity indices

To determine the mutational differences within and among the populations of hybrid parents by partitioning their variation, AMOVA was performed using the GenALEx version 6.5 Software. Based on the SNPs and sub-populations determined from the STRUCTURE results, an AMOVA estimation was carried out. Furthermore, to measure the genetic similarity of pair-wise genotypes from the parental inbred population, Phi-statistics (PhiPT) and the number of migrants (Nm value) were estimated as a genetic differentiation parameter. In addition, several genetic indices, such as number of effective sales (Ne), number of different titles (Na), Shannon Information Index (I), Diversity Index (h) and Unbiased Diversity (uh) were calculated using GenALEx version 6.5 Software.

### Linkage disequilibrium (LD)

The investigation of linkage disequilibrium (LD) between pairs of SNP markers across the genome was used to assess the squared allele frequency correlation (r^2^). This analysis focused on pairs of intrachromosomal SNPs with known genomic positions. The estimation was conducted using TASSEL v5.2.82 software. A sliding window approach was employed to explore the linkage disequilibrium (LD) between pairs of SNP markers spanning all seven chromosomes. Additionally, LD was computed as a full matrix for pairs of the same SNP markers within each chromosome using the TASSEL software. A graphical representation of the average pattern of genome-wide LD decay over genetic distance was constructed by plotting the r^2^ of alleles against the corresponding genetic distance between markers as a scatter plot. Finally, the non-linear regression was fitted to obtain the LD decay curve as described by [22], modified by [23] was produced in R (R core team, 2018) using the ’ggplot2’ package.

### Field evaluation

A set of 87 parental lines (35 B- and 51 R-lines derived from the earlier mentioned set of lines and one additional R-line) were evaluated in three different trial groups, namely PT-1, 2 and 3. Each of these sets of hybrid parental lines (PT-1:28 lines, PT-2:36 lines and PT-3:36 lines) was evaluated with separate blocking of B- and R-lines in each replication to avoid the suppressive effect of R-lines over B-lines (as R-lines are taller than B-lines). Thirteen of such experimental trials (set × location × year) (Table 1) were planted during the rainy season of 2020 and 2021 at five experimental stations (locations) in the drought-prone ecology of North-western India. The experimental sites included Jodhpur (26°27’N), Durgapura (26°84’N), Bikaner (28°09’N), Malakhera-Alwar (27°39’N) in Rajasthan and Hisar (29°15’N) in Haryana (S2 Table). In different parental trials, a Randomized Complete Block Design (RCBD) and Alpha Lattice design were implemented, each comprising two replications. Each parent trial was conducted in at least three environments (two replications × three to four environments/location×year) (Table 1). All parental trials had four to six common parental checks (ICMB 92777, ICMB 98555, ICMB 98222, H77/83-2, ICMR 14888, and ICMR 1202). Each entry was planted in a plot size of two rows of 4-meter length with an inter-row spacing of 0.5 to 0.6 meters per the local practice, and plants were spaced 15 centimetres apart in a row (S2 Table).

**Table 1:** Summary of experimental trials conducted during Rainy 2020 and 2021 at five locations in drought-prone environments of North-western India.

Trial Name	Number of Entries	Environment (Location*Year)	Replication	Experimental Design
*PT-1	24 + 4	7	2	^$^RCBD
PT-2	32 + 4	3	2	RCBD
PT-3	32 + 4	3	2	Alpha- Lattice (6 x 6)

*PT = Parental trial and $RCBD = Randomised complete block design.

### Trait measurement

Agro-morphological traits, including grain yield and component traits, were recorded. Plant height was measured from the base of the stem to the tip of the panicle of the main tiller at the time of harvest on five random plants of each entry in each replication. While for days to 50% flowering (DB), days to maturity (DTM), grain yield (GY), panicle yield (PY) and dry fodder yield (DFY) were measured on a plot basis. The DB recorded when 50% of the plants showed stigma emergence. The panicles from each plot for each entry were harvested and then dried under sunlight for (4–7 hours/day) for 10–15 days. After obtaining the panicle dry weight, the panicles were threshed, and the grain yield was recorded. After harvesting all panicles, the biomass of each plot was sundried for 12–15 days, and data for stover yield/DFY for each plot were recorded. The plot yield for grain, panicle and dry fodder was converted into kilograms per hectare. The panicle harvest index (PNHI) was calculated as the ratio of GY to PY and was expressed as a percentage. The PY and DFY were added to obtain the biological yield, and then Grain Harvest Index (HI) was calculated for each plot as the ratio of GY to the biological yield and multiplied by 100. From each plot, approximately 100–150 grams of grain samples were collected to estimate the grain iron (Fe) and zinc (Zn) content and for an additional 1000 grain weight (TGW) estimation. The grain iron (Fe) and zinc (Zn) densities were measured using an energy-dispersive X-ray fluorescence spectrometry machine (ED-XRF), model X-supreme 8000, from OXFORD [24]. After the ED-XRF analysis of the grain sample, random samples of 1000 grain seeds from each entry were counted with the help of an R-25+ seed counter from the Data Technologies Company (from Israel), and TGW was recorded.

### Statistical analysis of field data

RCBD and Alpha lattice designs were laid out in different trials, and combined analysis for multi-environment trials was performed using an unweighted two-stage analysis approach to estimate surrogate values of phenotypes. In the first stage, BLUEs (Best Linear Unbiased Estimators) and residuals were estimated from the environment-wise analysis and in the second stage genotype means (BLUEs) and environments were considered as factors and performed combined analysis [25] was performed as follows:

$$Y_{ijkl}=\mu +g_{i}+t_{j}+r_{jk}+b_{jkl}+e_{ijkl}$$ Eq (1)

where *y*_*ijkl*_ is the grain yield of the *i*th genotype in the *j*th trial in the *l*th block within the *k*th replication, μ is the intercept, g_*i*_ is the effect of the *i*th genotype, t_*j*_ is the effect of the *j*th trial, r_*jk*_ is the effect of *k*th replication in the *j*th trial, b_*jkl*_ is the nested effect of *l*th block in *k*th replication within *j*th trial, and e_*ijkl*_ is the error of yijkl. Replication and block were treated as random effects and genotype as fixed effects to estimate BLUEs. To estimate the repeatability, all factors were considered random.

The adjusted genotype means (BLUEs) were calculated from the second stage by using the following model:

$$Y_{ij}=\mu +g_{i}+u_{j}+e_{ij}$$ Eq (2)

Where y_ij_ is the grain yield of the *i*th genotype in the *j*th environment, μ is the general mean, g_*i*_ is the effect of the *i*th genotype u_*j*_ is the effect of *j*th environment and eij is the error of y_*ij*_. To estimate the BLUEs across environments, genotypes and environments were treated as fixed and random effects, respectively. All linear mixed models were implemented using the ‘lme4’ R package [26].

### D^2^ statistics

The estimated mean values of the hybrid parents from the second-stage analysis of the 11 morphological traits were used to assess the phenotypic diversity. Of the 87 parental lines evaluated for phenotypic traits, genotyping data were available for 84 lines (34 B-lines and 50 R-lines). For all 84 genotypes, the generalized Mahalanobis distance [27] between all pairs of rows (genotypes) in a data frame with respect to a covariance matrix was calculated. The 84 hybrid parent means were grouped into different clusters based on Mahalanobis D^2^ distance using the Ward’s method [28] using the ’biotool’ R package [29].

### Joint analysis of phenotypic and genotypic data

A joint analysis was conducted using a combination of phenotypic and genotypic dissimilarity matrices for those 84 lines. The genotypic dissimilarity matrix was constructed by calculating Roger’s distance using BIO-R software [19]. While the phenotypic distance matrix was derived using D^2^ statistics. The Mantel test [30] was employed to evaluate the relationship between genetic distances among genotypes based on Roger’s distance and Mahalanobis D^2^ distance using GenALEx version 6.5 Software. Phenotypic and genotypic hierarchical clusters were compared using the tanglegram function in the ’dendextend’ R package [31].

## Results and discussion

### Distribution of SNP markers across the genome

In the present study, 109 pearl millet hybrid parental lines genotyped using GBS had 7,22,672 raw SNPs, and 16,472 SNPs were considered for the final analysis after applying the filtration criteria. The SNPs were distributed across all the seven chromosomes, chromosome 2 had the highest number of SNPs (3227), followed by chromosome 1 (2737), while chromosome 6 had the lowest number of SNPs (1740) (Fig 1 and S3 Table). The distribution of high-quality SNPs was observed across the seven pearl millet chromosomes, exhibiting a distinct concentration in the telomeric regions compared to that in the pericentromeric regions (Fig 1). This phenomenon is hypothesized to be influenced by factors such as low recombination rates, reduced gene density, and/or the limited presence of restriction sites for enzymes proximal to the centromere [32]. Marker density throughout the genome ranged from 0 to 50 SNPs per Mega base pairs (Mb), with an average of 11 SNPs per Mb. The average distance between two SNP markers was found to be 95 kb (S3 Table). However, the distribution of SNP-to-SNP distances showed skewness: 42% of the marker-to-marker distances were less than 1 kb, whereas 78% were less than 100 kb. This SNP density comparatively lower than earlier reports where they had reported 35 SNPs per Mb [32] and 48 SNPs per Mb [33].

**Fig 2:**
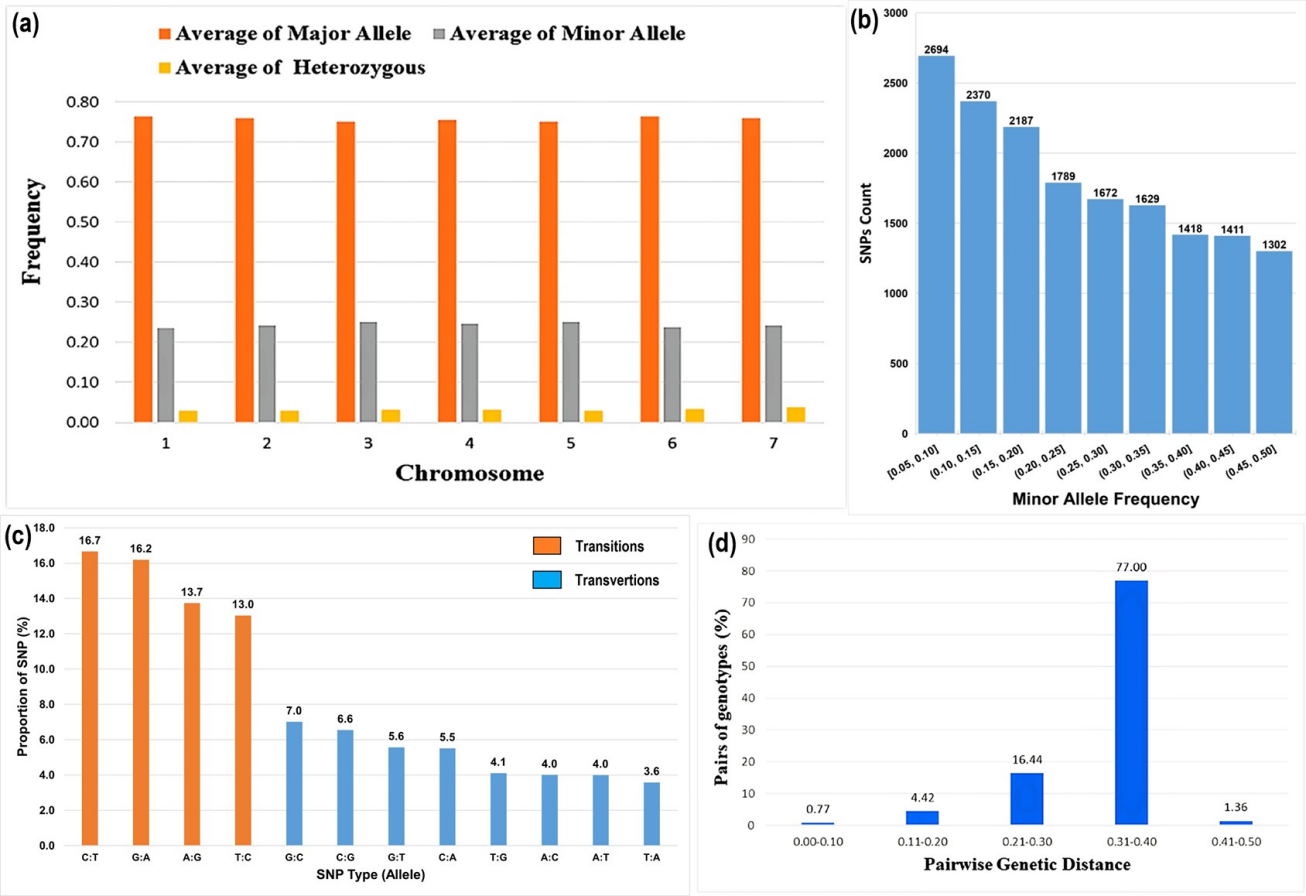
(a) Frequency distribution of the 16,472 polymorphic SNPs across seven chromosomes; (b) Frequency distribution of the minor alleles for each SNP markers scored in a population sample of 109 genotypes; (c) Transition and transversion mutations of GBS-SNPs detected among 109 pearl millet hybrid parental lines; (d) Identity-by-state (IBS) based genetic distance between pairs of hybrid parental lines.

Quality parameters of the 16,472 SNP markers for the hybrid parental lines are presented in S4 and S5 Tables. Minor allele frequency (MAF) ranged from 0.05 to 0.5 with a mean value of 0.24 and had heterozygosity values ranged from 0.001 to 0.17 with a mean value of 0.031. The major allele frequency (MaF) ranged from 0.5 to 0.95 with an average value of 0.76, and the gene diversity values varied from 0.062 to 0.65 with a mean of 0.568 (Fig 2A and 2B and S4 and S5 Tables).

SNPs were classified based on nucleotide substitutions into transitions (C↔T or A↔G) or transversions (A↔C, C↔G, A↔T, and G↔T). The analysis revealed a prevalence of transition mutations (9,823, 59.63%) compared to transversion mutations (6,649, 40.37%), resulting in a transition/transversion ratio of 1.48 (Fig 2C). Overall, A/G transitions exhibited the highest frequency, whereas A/T mutations were the least common among the detected mutation types. The frequencies were comparable between the A/G and C/T transitions, and among the four transversions, C/G had the highest frequencies. The identification of a higher occurrence of SNPs with transition substitutions (57%) compared to transversions aligns with earlier genome-wide SNP discovery investigations in agricultural crops [34,35]. This phenomenon, termed as ’transition bias,’ has been noted in rice [36] and maize [36,37]. The preference for transitional mutations over transversions is attributed to their conformational advantage in instances of mispairing and their better tolerance during natural selection. Transitions are more likely to preserve protein structure than transversions [38].

The pair-wise genetic distance values based on Identity-by-State (IBS) inbred lines varied from 0.007 to 0.417, with an overall average of 0.337 (S6 Table). Among all pairs of lines, seventy-seven per cent showed a 30–40% allele difference, while 16% of the pairs showed an allelic difference of 20–30% (Fig 2D), indicating the presence of significant genetic diversity among the hybrid parental lines, even though some of the lines had involvement of common parental lines in their pedigree. Lines ICMB 04999 (B2) and MSR 22 (R38) had the highest genetic diversity (0.417), suggesting that these two inbred lines had the maximum divergence. While lines ICMB 10222 (B7) and ICMB 11777 (B27) showed the lowest genetic diversity (0.0067), which could be due to the sharing of common parent 843B in their parentage/pedigree. Nucleotide diversity (π) and Tajima’s D statistics for the parental population are presented in Table 2. Nucleotide diversity in the population ranged from 0.307 to 0.371, with an average of 0.337. The Tajima’s D value ranged from 2.078 to 3.210, with a mean value of 2.607. Tajima’s D statistics were used to evaluate deviation from the expected patterns of neutral variations, and a positive value of Tajima’s D suggests an excess of common variation in a region due to balancing selection, or sudden population contraction, or population subdivision. In comparison, a negative value indicates an excess of rare variation due to selective sweep or population expansion after a recent bottleneck, and a zero value suggests no evidence of selection [39]. In the current study, the high positive value of Tajima’s D revealed a significant deviation from neutral evolution (D = 0), and the presence of rare alleles at low frequencies in a population. This indicates that the populations may have undergone through balancing selection with the aim of maintaining variation among the population during their breeding progress. Similar positive Tajima’s D values have been observed in different sets of pearl millet inbred lines [32] and wheat germplasms [40]. Positive values for Tajima’s D suggested the presence of rare alleles at low frequencies.

**Table 2:** Summary statistics for 109 inbred lines based on 16472 SNP markers.

	MaF	GD	He	MAF	PiPerBP (π)	Tajima’s D
Minimum	0.5	0.007	0.001	0.05	0.307	2.078
Maximum	0.95	0.417	0.17	0.5	0.371	3.210
Mean	0.76	0.337	0.031	0.24	0.337	2.607

MaF = Major allele frequency, GD = Pairwise genetic distance, He = Heterozygosity, MAF = Minor allele frequency, PiPerBP (π) = Nucleotide diversity.

### Bayesian model-based population stratification

**Fig 3:**
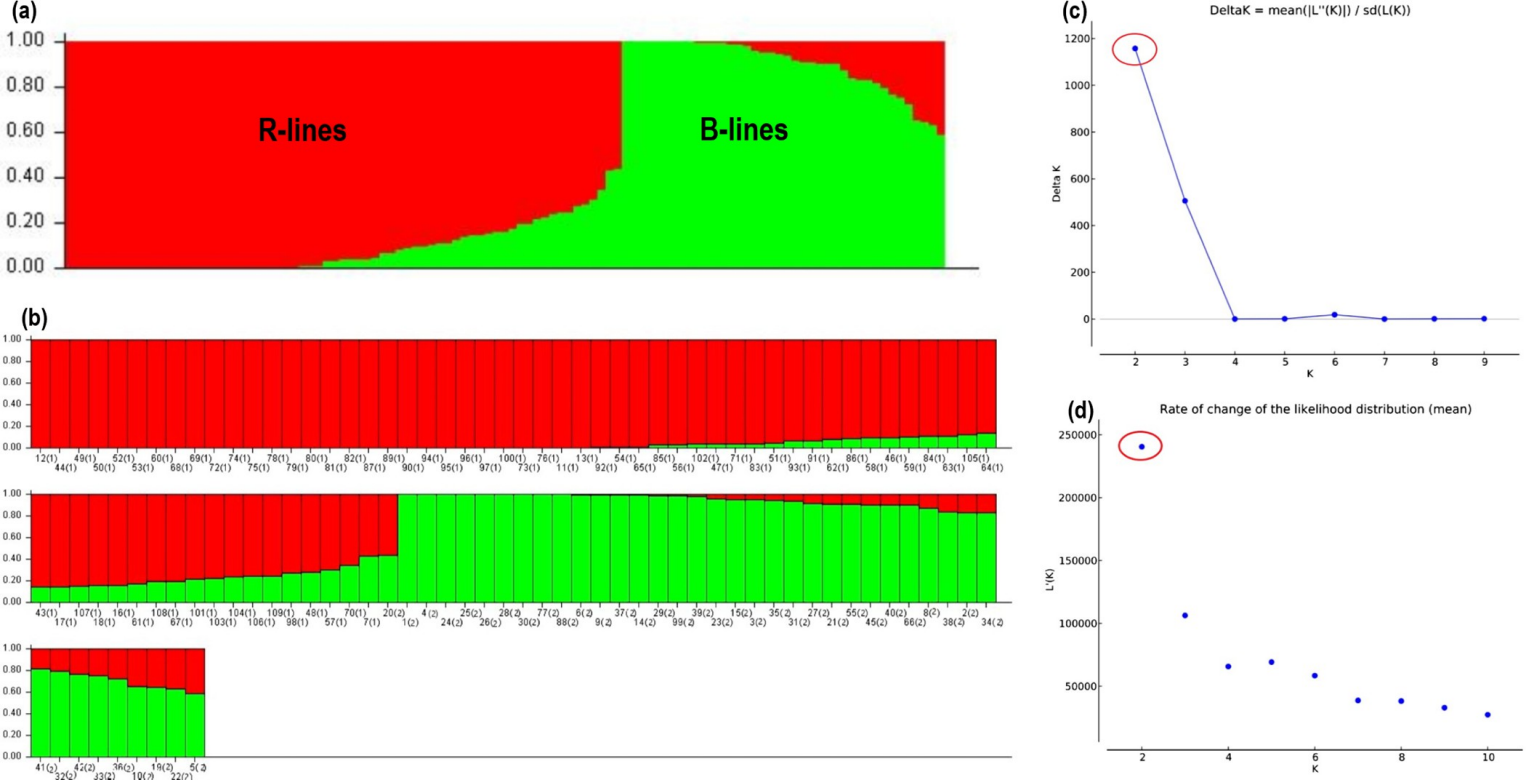
Population structure analysis of 109 hybrid parental genotypes: (a) estimated bar plot of the population sorted by kinship matrix; (b) graphical representation of 109 hybrid parental lines using 16,472 markers for K = 2;(c) delta (Δ) k for different numbers of sub-populations (k); (d) rate of change of likelihood distribution (mean) |Ln’’(K)| using the Evanno method.

Bayesian model-based (STRUCTURE) analysis of the SNP marker showed the highest Δk peak at k = 2 based on the Evano criterion (Fig 3C and 3D), which indicating the presence of two subpopulations (Q1 and Q2) (Table 3). The sub-population (Q1) comprised 61.6% of the inbred population (67 lines), whereas the sub-population (Q2) contained 38.4% of individuals (42 inbred lines). Q1 contained most of the R-lines (pollinators), while Q2 possessed all B-lines except for one R-line (Fig 3A and 3B). Individuals with a score for clusters membership coefficient more than 0.80 are considered as pure, while those who scored less than 0.80 was considered as admixture [41,42]. In the case of the R-lines cluster (Q1), 67 accessions were identified, out of which 58 were pure and nine were admixtures. However, in the B-lines cluster (Q2), out of 42 accessions, 32 were pure and 10 were admixture (S7 Table). Although there were few admixtures across sub-populations, but the majority of lines formed two distinct clusters: B- and R-lines. One of the R-lines, namely ICMR 14888 (R69), demonstrated an inferred ancestry within the Q2 sub-population (B-lines). This admixture may be resulted because of cross-breeding and common breeding history between B- and R-lines. This might be due the fact that sometimes lines from other groups had been used as a source of new alleles/traits which were unavailable in the respective group [6,43]. The mean fixation index (Fst) was 0.18 and 0.497, while the expected heterozygosity was 0.315 and 0.203 for sub-populations Q1 and Q2, respectively (Table 3). The allele frequency divergence (net nucleotide distance) between the two sub-populations was 0.137. The population structure clearly showed two significant groups, the B- and R-lines, with some admixtures in their respective sub-populations. Earlier studies [43,44] also reported the existence of two sub-groups, one each for B- and R-lines, in two sets of populations with few admixtures.

**Table 3:** Results of Model-based Bayesian analysis for 109 inbred lines based on SNP markers.

Population	Fixation Index (Fst)	Expected Heterozygosity*	No. of Individuals	Proportion of membership	Net nucleotide distance^$^
Q1 (R-lines)	0.1802	0.315	67	0.616	0.137
Q2 (B-lines)	0.4973	0.203	42	0.384	0.137

* Average distances (expected heterozygosity) and ^$^Allele-freq. Divergence among populations (net nucleotide distance).

### Grouping of the genotypes based on their genetic relatedness and principal coordinate analysis

**Fig 4:**
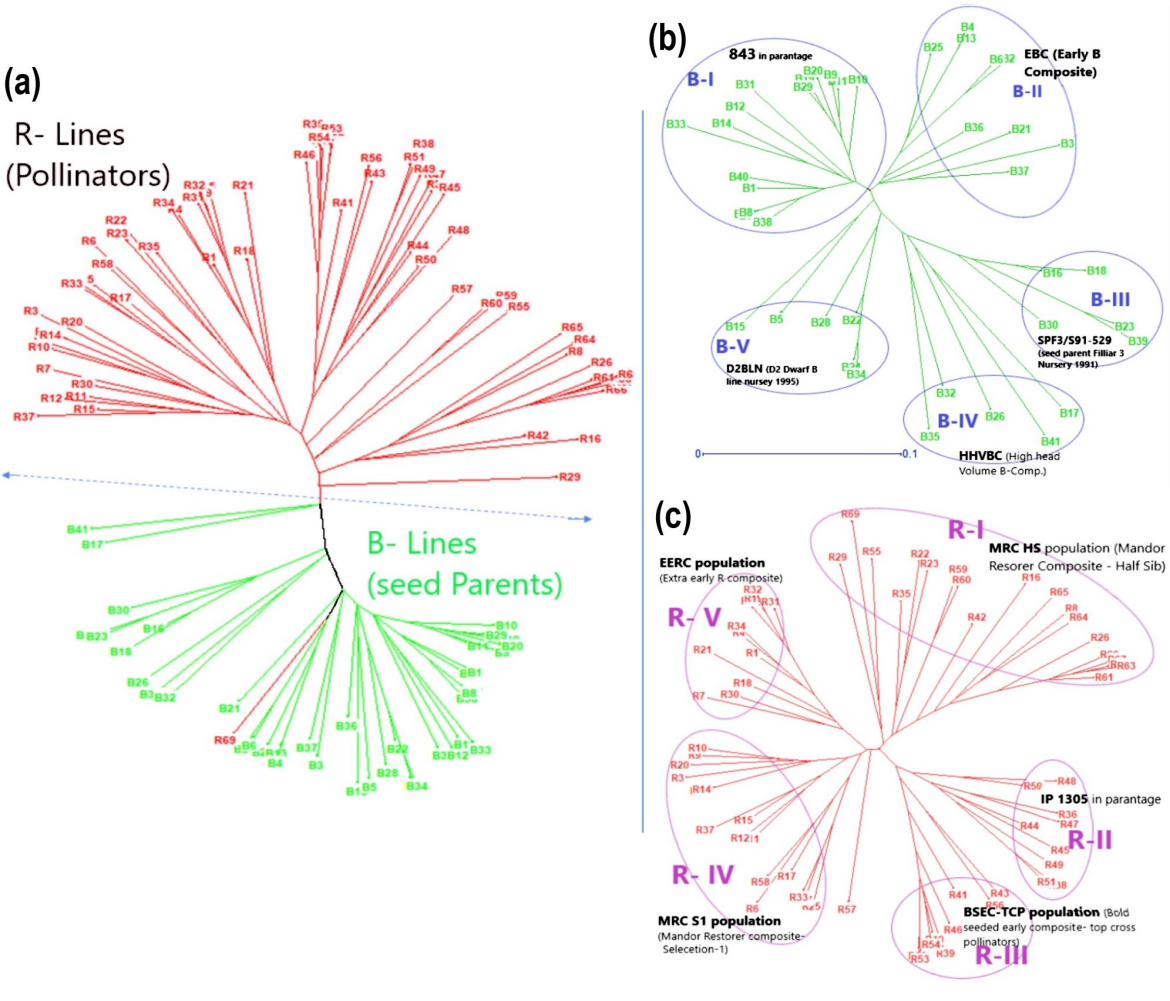
Unweighted neighbor-joining (NJ) tree showing clustering pattern and relatedness among hybrid parental lines based on GBS-identified ed SNPs (Nei’s Distance): (a) tree for 109 hybrid parents (41 B-lines and 68 R-lines), (b) tree for 41 B-lines, and (c) tree for 68 R-lines #Green- B lines (seed parents) and Red- R lines (pollinators).

The Nei’s genetic diversity (GD) for all 109 genotypes was estimated, and pair-wise genetic distance ranged from 0.003 to 0.324 with a mean value of 0.243. Compared to the B-line (0.165), the R-line possessed a higher mean genetic diversity (0.236). Among the B- and R-line groups, the R-line showed higher genetic dissimilarity (maximum 0.319) than the B-line pairs (maximum 0.262). The genetic dissimilarity between the B- and R-groups ranged from 0.117 to 0.324 (S8 Table and S1 Fig). The lowest GD was observed for pair ICMB 10222 and ICMB 11777 (0.003), which might be due to having 843B as a common parentage, while in the case of R-lines HPR4 and HPR7 showed the lowest GD as they had a common ICTP8203 parent in their parentage. However, the highest GD was observed between ICMB 04999 and MSR 22 (0.324); these results were similar to those of IBS. Based on Nei’s distance, a neighbor-joining (NJ) tree was generated, which clearly showed two major sub-populations, B-lines and R-lines, which was the same as the clustering pattern observed in the STRUCTURE results (Fig 4A). One of the R-line (R69), viz., ICMR 14888, was found grouped in the B-lines cluster and similarly observed in case of population structure. This might be due to the involvement of a population ICMS 8511, which was a founder parent in the breeding of some seed and restorer lines.

Furthermore, 41 B-lines were found grouped into five clusters: 16 B-lines in B-I (39%), nine in B-II (22%), five in B-III (12%), five in B-IV (12%) and six B-lines (15%) in B-V (Fig 4B). In cluster B-I (14 out of 16), had 843B was a common parent in their parentage which might have led to the formation of this cluster. In the case of the B-II cluster, most of the lines had arid type, extra-early and early material backgrounds in the parentage, such as B- composites such as EBC (Early B-line composite), HTBC (High tillering B-composite) and ARD [*Iniadi* germplasm accession ARD (Appa Rao, Rai and Djaney)]. In the case of the B-III cluster, all lines had SPF3/S91-529 (seed parent filial-3 nursery from summer 19991) in the pedigree. However, in cluster B-IV, two out of five lines had a high head volume B composite (HHVBC) in their parentage. Furthermore, cluster B-V had mostly D2 dwarf lines which might have been grouped together due to the involvement of D2BLN/95-93 (D2 Dwarf B Line Nursery 1995 Entry No 93) in their parentage (S9 Table).

Similarly, R-lines were grouped into five clusters: 20 R-lines in R-I (29%), nine in R-II (13%), nine in R-III (13%), 18 in R-IV (27%) and 12 R-lines (18%) in R-V (Fig 4C). Most of the R-lines in cluster R-I had MRC-HS (Mandor Restorer Composite Half sibs) in their parentage, whereas in the case of R-II cluster all the R-lines had IP 1305 germplasm accessions as a common parent in their parentage. In cluster R-III, seven out of nine lines had BSEC-TCP (bold seeded early composite- top cross pollinators) in their parentage and most of the lines with MRC S-1 (Mandor Restorer Composite Selection generation-1) and MRC-HS were found grouped in cluster R-IV (S9 Table). These results showed that the clustering patterns in the seed and restorer lines were associated with common parentage in the lines present in the respective clusters.

In the present study, the clustering pattern clearly delineated most seed parents (B-lines) and pollinators (R-lines) into separate groups. Similar findings were reported based on SSR markers where two separate major sub-populations for the B- and R-line groups were formed [6]. In addition, based on SNP markers, a similar clustering pattern was observed in the high-density [45] and mid-density panels [43]. The existence of B- and R-line in the two groups can be explained by the breeding strategy of the pearl millet hybrid breeding program. The clear-cut separate trait-specific breeding approach was adopted while utilising germplasm during the development of B-lines and for R-lines, and also because the involvement of separate breeding materials in the B- and R-line programs led to high genetic differences between B- and R-groups [44–49].

The selection of divergent lines based on genetic distance can be helpful for identifying heterotic cross combinations [50]. The twenty-five most diverse pairs within the B- and R-line groups were identified and which can be utilized for line development (S10 Table). Similarly, 25 genetically divergent pairs between the B- and R-lines were identified, which could utilized as potential heterotic cross combinations (S10 Table). Previous studies investigating the relationship between genetic distance and heterosis have indicated that genetic distance is a key factor in predicting heterosis [45,51–53].

**Fig 5:**
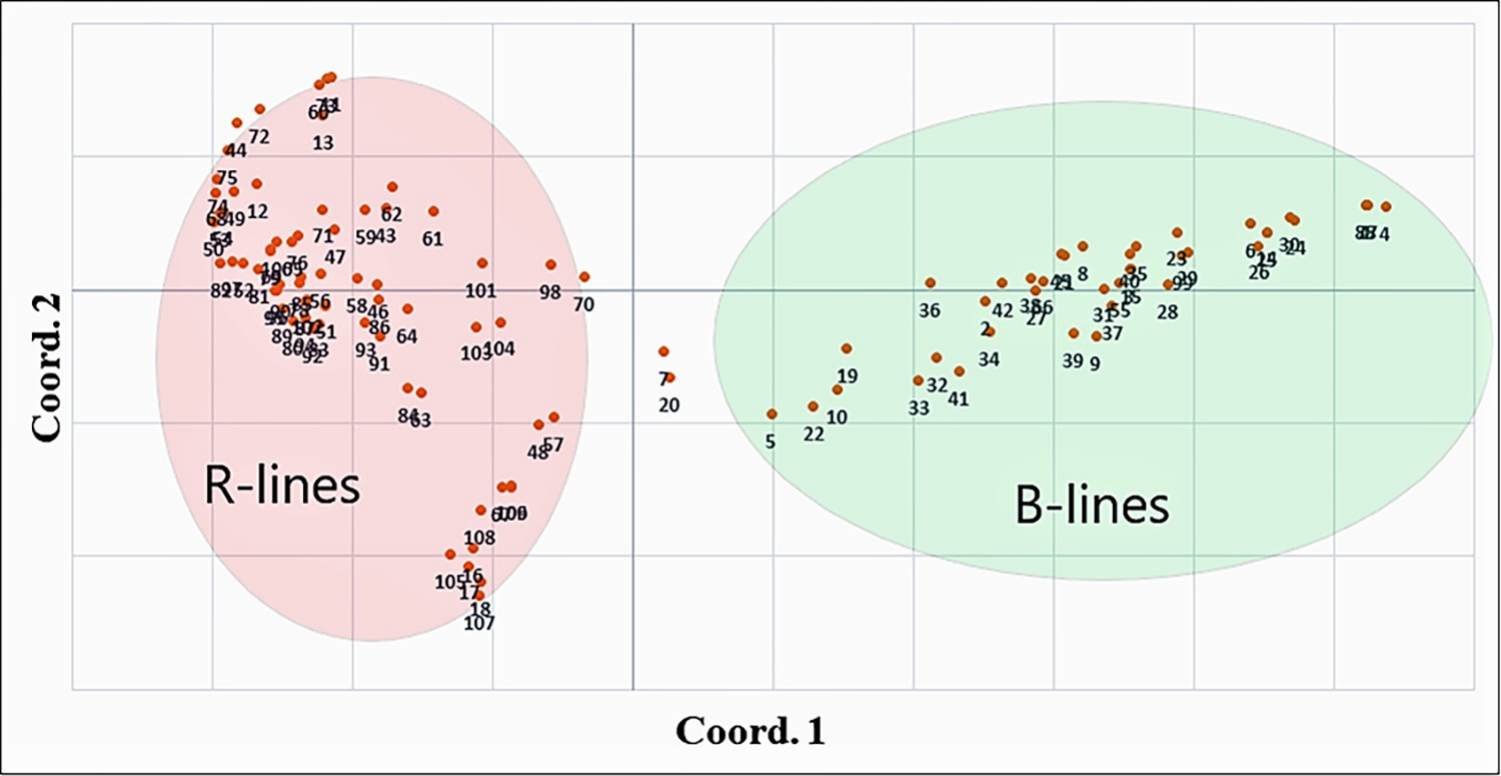
Principal coordinate analysis (PCoA) for seed and pollinator parents based on genetic distance estimated by using GBS-identified SNP.

PCoA was performed for 16,472 SNP markers based on Nei’s distance from the 109 hybrid parents. The first three principal coordinates revealed 15.32% of the total genetic variation, and the first two axes were used together for plotting all accessions, which explained 12% of the total variation. Two-dimensional plotting of genotypes based on coordinates was performed for the first and second principal coordinates, with 8.07 and 3.96% of the total genetic variation, respectively (S11 Table). PCoA is helpful in summarizing and representing the relationship between the number of genotypes in a simple Euclidean space. All genotypes were broadly grouped into two major clusters: B- and R-lines. These results are in agreement with the results of STRUCTURE analysis of genetic diversity-based clustering patterns, where hybrid parental lines were broadly classified into two sub-groups (Fig 5). In a previous study, principal component analysis (PCA) was performed to assess the diversity among the 373 inbred parental lines, which again showed two major groups plotted in the first three PCs [43].

### Genetic differentiation based on molecular variance and allelic pattern across the population

The Analysis of molecular variance (AMOVA) was performed to estimate genetic differentiation among B and R population sub-groups, revealing that 7% of the total variation was among sub-populations, i.e., between seed (B-lines) and pollinator (R-lines) parents, whereas the remaining 93% of the variation was within the sub-populations (Table 4). The PhiPT (analogue to Fst) value for testing the statistical significance of the estimated population structure was 0.069 (with a maximum value of 0.995 and minimum value of Phi’PT of 0.07) and an associated permutation p-value <0.01, which indicates moderate genetic differentiation (Table 4). The phi statistics (PhiPT) is a modified form of Wright’s Fst, which estimates the relative portion of variation between groups/populations to overall/total genetic variation. PhiPT value of ≤ 0.05 indicated negligible genetic differences, while values between 0.06 to 0.15 are considered moderate, values between 0.16 to 0.25 are considered high differentiation, and >0.25 indicates great genetic difference [54]. The results of the AMOVA indicated the presence of significant diversity between the two sub-populations (~7%) compared to the high amount of genetic diversity within the sub-population. Earlier studies have also reported the same where variation within the population (intra-population) was higher than that between populations [43,53,55]. The most probable explanation for high variation within populations might be due to the frequent selection for agronomically important traits during breeding progress [40].

**Table 4:** Analysis of molecular variance using 16,472 SNPs for two subpopulations of 109 hybrid parental lines.

Source	df	SS	MS	Est. Var.	%
Among Pops	1	178.78	178.78	2.77	7
Within Pops	107	3977.61	37.17	37.17	93
Total	108	4156.39		39.94	100
Stat	Value	P (rand > = data)			
PhiPT	0.069	0.001			
Nm (Haploid)	6.715				

% = percentage of molecular variance.

The extent of genetic diversity between and within a population is a function of the rate of gene flow within the population. To quantify the level of gene flow among the population (between the B- and R-lines), the number of migrants (haploid Nm) was estimated. The value for Nm (haploid) was high (6.715), indicating a high gene exchange among the sub-populations (Table 4). Nm (haploid) values less than 1 indicated limited gene exchange among the populations [56]. The higher level of genetic variation residing within a population than among populations indicates a good amount of genetic variation at the population level to select diverse and promising hybrid parental lines. This will be helpful in developing a well-characterized population to select parents contributing to good adaptation, persistence, and yield [57].

Based on SNP markers, different diversity indices such as Shannon’s diversity index (I), and unbiased diversity (uh), were used to assess genetic diversity among the 109 hybrid parental lines. The Shannon’s diversity index for B-lines (0.49), R-lines (0.615) and the overall population (0.557) was observed (Table 5). Compared with B-lines (2.494), the R-line group (2.857) had a higher number of different alleles (Na) and the B-line group possessed a lower number of effective alleles (Ne) (1.52) than the R-line group (1.584). The diversity (h) and unbiased diversity (uh) were higher in the R-line population (h = 0.366 and uh = 0.371) compared to the B-line population (h = 0.298 and uh = 0.305). The mean values of Na, Ne and h for the overall population were 2.676, 1.557 and 0.332, respectively (Table 5). The percentage of polymorphic loci per population was 96.29% for B-lines and 99.74% for R-lines, with an average of 98.02% (Table 5). High values of these indices indicated the presence of high genetic diversity [58,59]. These results suggested that, among the two different groups, the R-line sub-population was more genetically diverse. In addition, higher genetic diversity and a greater number of different alleles were detected in R-lines which might be due to the involvement of more diverse germplasm during the breeding of R-lines, which has been reported earlier [6,45,46,48].

**Table 5:** Mean of different Genetic diversity indices for the two estimated population structures for 109 inbred lines based on 16472 SNP markers.

Genetic Diversity Parameters	B-lines	R-lines	Overall
% of polymorphic loci	96.29%	99.74%	98.02%
Expected Heterozygosity (He)	0.320	0.347	0.334
Observed Heterozygosity (Ho)	0.028	0.038	0.031
No. of Different Alleles (Na)	2.494	2.857	2.676
Number of effective alleles (Ne)	1.522	1.584	1.557
Shannon Diversity Index (I)	0.499	0.615	0.557
Diversity (h)	0.298	0.366	0.332
Unbiased Diversity (uh)	0.305	0.371	0.338

Ne = 1 / (Sum pi^2), I = -1* Sum (pi * Ln (pi)), h = 1—Sum pi^2, uh = (N / (N-1)) * h; Where pi is the frequency of the ith allele for the population and Sum pi^2 is the sum of the squared population allele frequencies.

### Linkage disequilibrium (LD)

**Fig 6:**
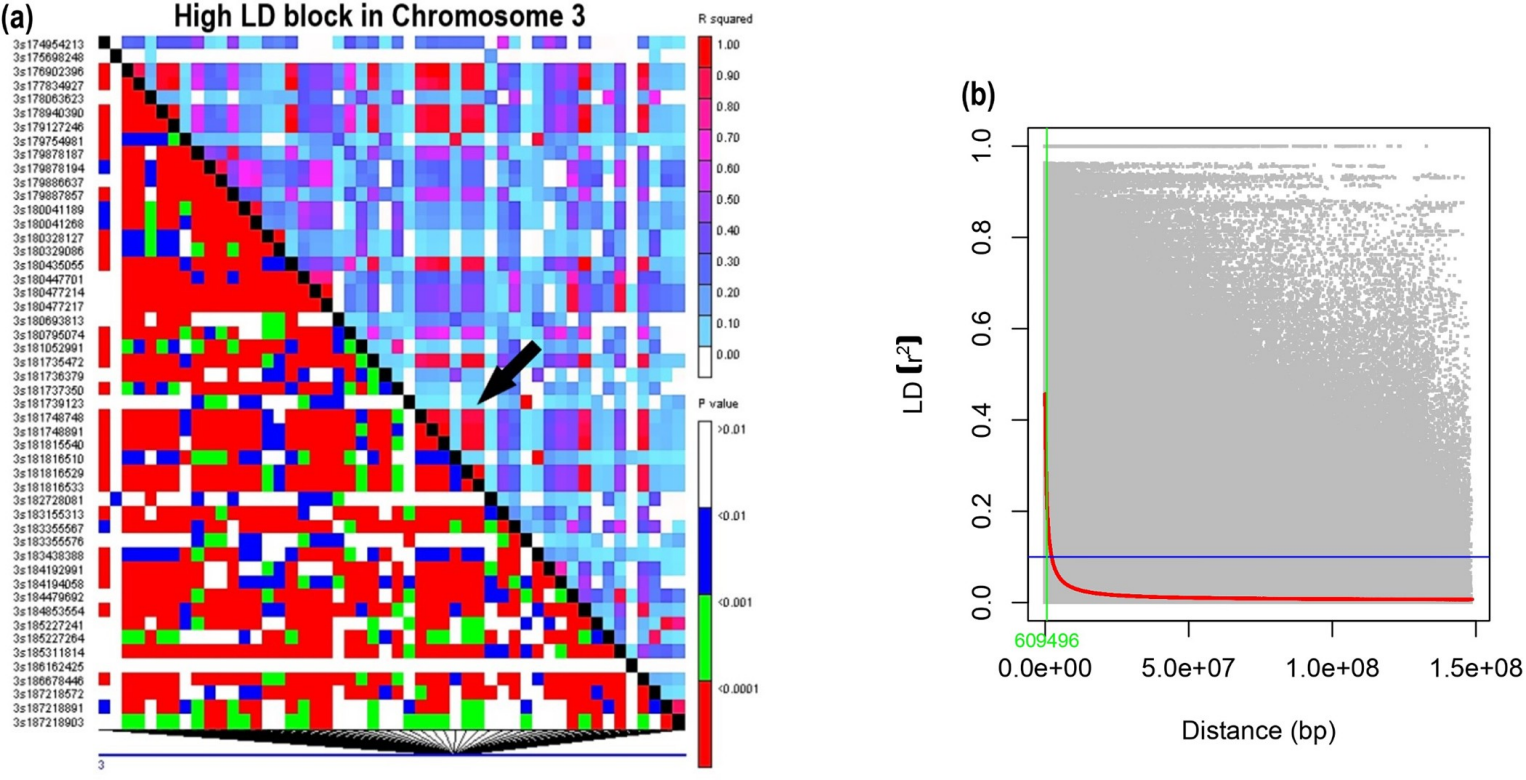
Linkage Disequilibrium pattern based on SNP identified via GBS (a) Triangle plot representing LD among SNPs for Chr 3 regions which showed a high LD pattern, Pairwise LD values were plotted on the X- and Y- axes; the above diagonal represents the squared correlation coefficient (r^2^), and the below diagonal represents the corresponding P-value; (b) Linkage Disequilibrium Decay (LDD) plots which show a decline in r^2^ between SNP pairs presented as a function of physical distance in pairs for the entire genome.

Among all the seven chromosomes, chromosome (chr) 3 showed the highest LD, followed by chromosomes 7 and 2. At the same time, the lowest LD was observed for chr 1, followed by chr 4 (Figs 6A and S2). Genome-wide LD signals help to understand the history of changes in population size and patterns of gene exchange [60]. In our study, the regions of high to low LD were observed on various chromosomes (S2 Fig). The highest LD was observed for chromosome 3, which was attributed to the low level of recombination events and the fixation of alleles (more conserved sites). These results were consistent with the previous study [43]. The uniform distribution of high LD regions along the chromosomes suggests that these loci could possess single or multiple genes of interest, which are of agronomic importance and are under selection by several breeding cycles/programs [61].

The LD decay distance varied from 0.37 Mb to 2.7 Mb across all seven chromosomes. A rapid decline in the average pair-wise LD (r^2^) with increasing physical distance was observed. The r^2^ values were generally below 0.05. The average LD decay distance over all seven chromosomes in the entire panel with r^2^ < 0.1 was 0.609 Mb (609.5 Kb) (Fig 6B). The source of inbred lines and selection intensity during the breeding process can influence the LD decay distance. Thus, a higher value for LD decay distance is usually observed in inbred cultivars compared to diverse germplasm accessions/collections [23,62–64]. In our study, the average LD decay (0.609 Mb) is comparatively higher than that observed in the earlier study (3.5 Kb and 200kb) [32,33], which suggested comparatively slower LD decay in our set of hybrid parental lines, than in earlier reports. This might be because the number of markers and the population size plays a crucial role in the estimation of LD values; the smaller the number of markers, the larger would be the LD values and the increase in LD decay distance due to the low sample size [65]. Thus, the low sample size (109 genotypes) in the current study compared to earlier ones may have resulted in high LD decay in this study. A rapid decline in LD decay was observed across the genome and chromosomes (Fig 6). Also, different levels of breeding and population progression from diverse landrace to elite inbred lines can lead to different levels of LD decay [62]. Estimating LD decay is important in GWAS studies as it depicts the minimum number of markers required to efficiently cover the genome efficiently for mapping traits [66]. The present study observed a rapid decline in LD decay with increasing distance between markers for chromosomes 1 and 4 compared to chromosomes 3 and 7. Among the chromosomes in the panel, chr 1 showed the smallest distance (~0.37Mb), followed by chr 4 (~0.52 Mb), whereas chr 3 (~2.78 Mb) and chr 7 (~0.92Mb), which showed the largest distance (S3 Fig). These results indicate that more markers are required for chr 1 and chr 4 than for chr 3 and chr 7 for GWAS (genome-wide association studies), and similar results were reported earlier [32]. The high genetic distance among most of the pairs of lines, along with rapid LD decay, suggests the uniqueness of the majority of parental lines, which can be utilized in the breeding programs [66].

### Grouping of genotypes based on morphological traits

**Fig 7:**
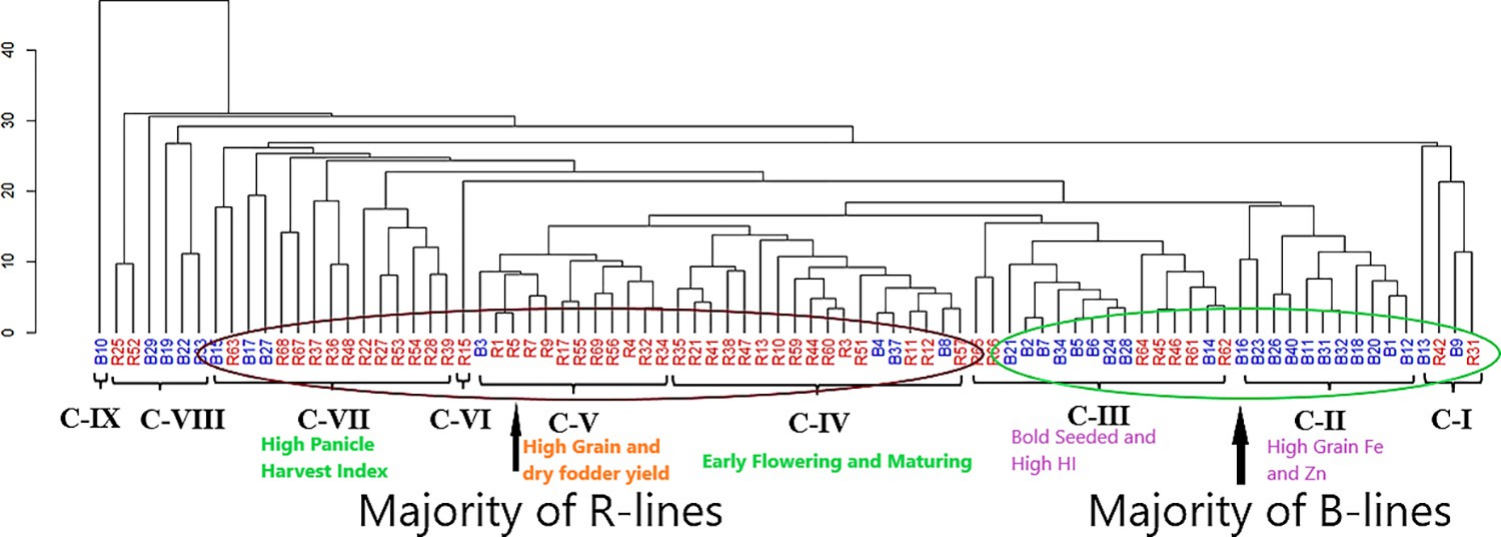
Dendrogram depicting a clustering pattern for relatedness among 84 hybrid parental lines based on morphological traits. # Blue labels- B lines (Seed parents) and red labels- R lines (Pollinators).

Multivariate analysis of the 84 parents based on phenotypic performance (S13 Table) was delineated into nine different clusters (Fig 7). Most of the seed parents grouped into three clusters (C-I, II and III); 65% of B-lines (22 out of 34) were found grouped. In contrast, the majority of pollinators (78%) (39 out of 50 R-lines) were found grouped in four clusters (C-IV, V, VI and VII). Cluster IV was the largest cluster with 18 lines with 3 B-lines and 15 R-lines, followed by Cluster III, cluster VII, cluster V, cluster II, cluster VIII, and Cluster I with 16, 15, 11, 6 and 4 parental inbred lines respectively. the remaining clusters, VI and IX, each had only one parental line each. The dendrogram clearly showed that most seed parents and pollinators could be grouped into two separate major groups.

**Fig 8:**
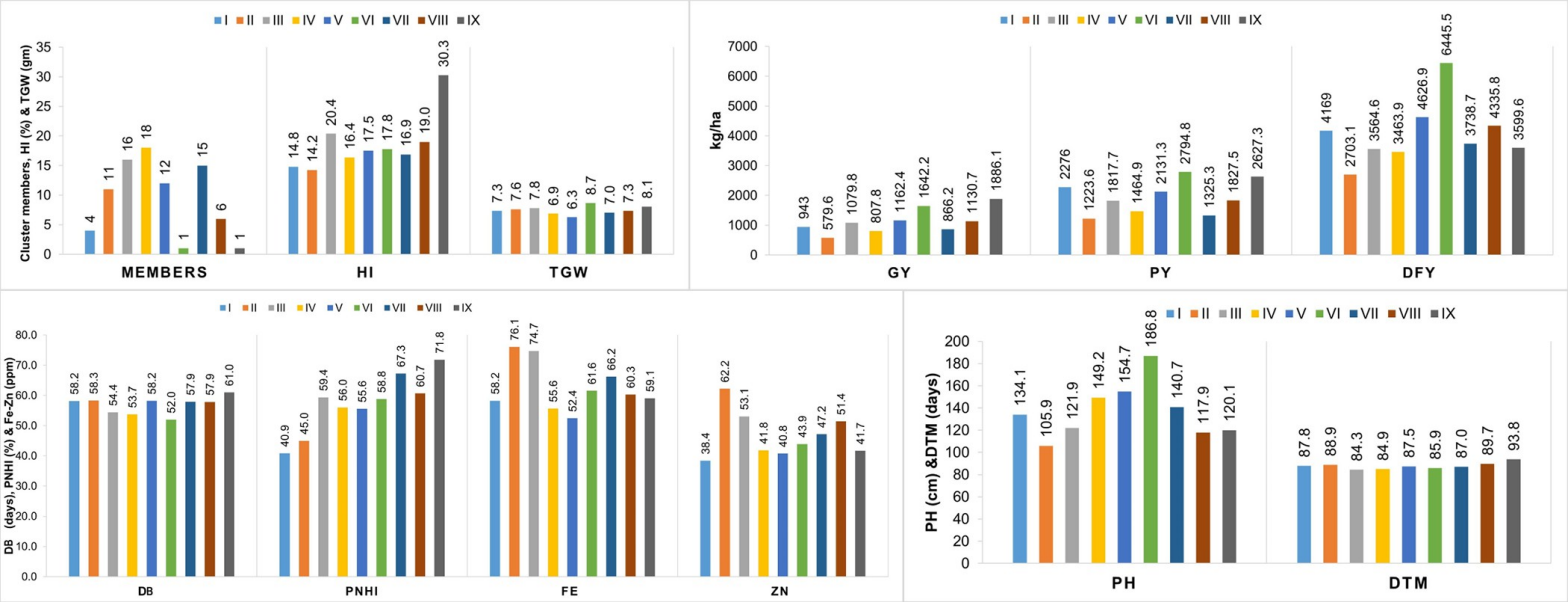
A graphical depiction of the average values for yield, grain quality, and yield component traits in 84 hybrid parental genotypes, based on phenotyping data from cluster analysis.

Cluster II was characterised by high grain Fe and Zn densities and had all B-lines in this group. Cluster III had a high cluster mean for 1000 grain weight (TGW), high HI and low DFY and GY values. The early flowering and maturing hybrid parents from cluster IV had the lowest mean values for DB and DTM (Fig 8 and S14 Table). Cluster V had the highest mean values for GY and DFY so that dual-purpose parents could be selected from these groups, while Cluster VII had the highest value for PNHI, which is desirable as it indirectly denotes better drought tolerance, because PNHI is an indicator trait for selecting tolerant genotypes under terminal drought conditions [67].

Critical examination of individual clusters revealed that most B- and R-lines were grouped into separate clusters. A similar clustering pattern for hybrid parents was observed in the case of [68]. This might be due to the reason that B-lines are bred for a specific set of traits, like short height (<100cm), early to medium maturity, high tillering, optimum grain and dry fodder yield, large seed size and a good GCA for yield [69,70]. At the same time, R-lines are bread for taller height (150–180 cm), early maturity, good pollen load (profuse pollen production), good tillering and relatively small grain size [70,71].

### Comparative grouping of hybrid parental lines

**Fig 9:**
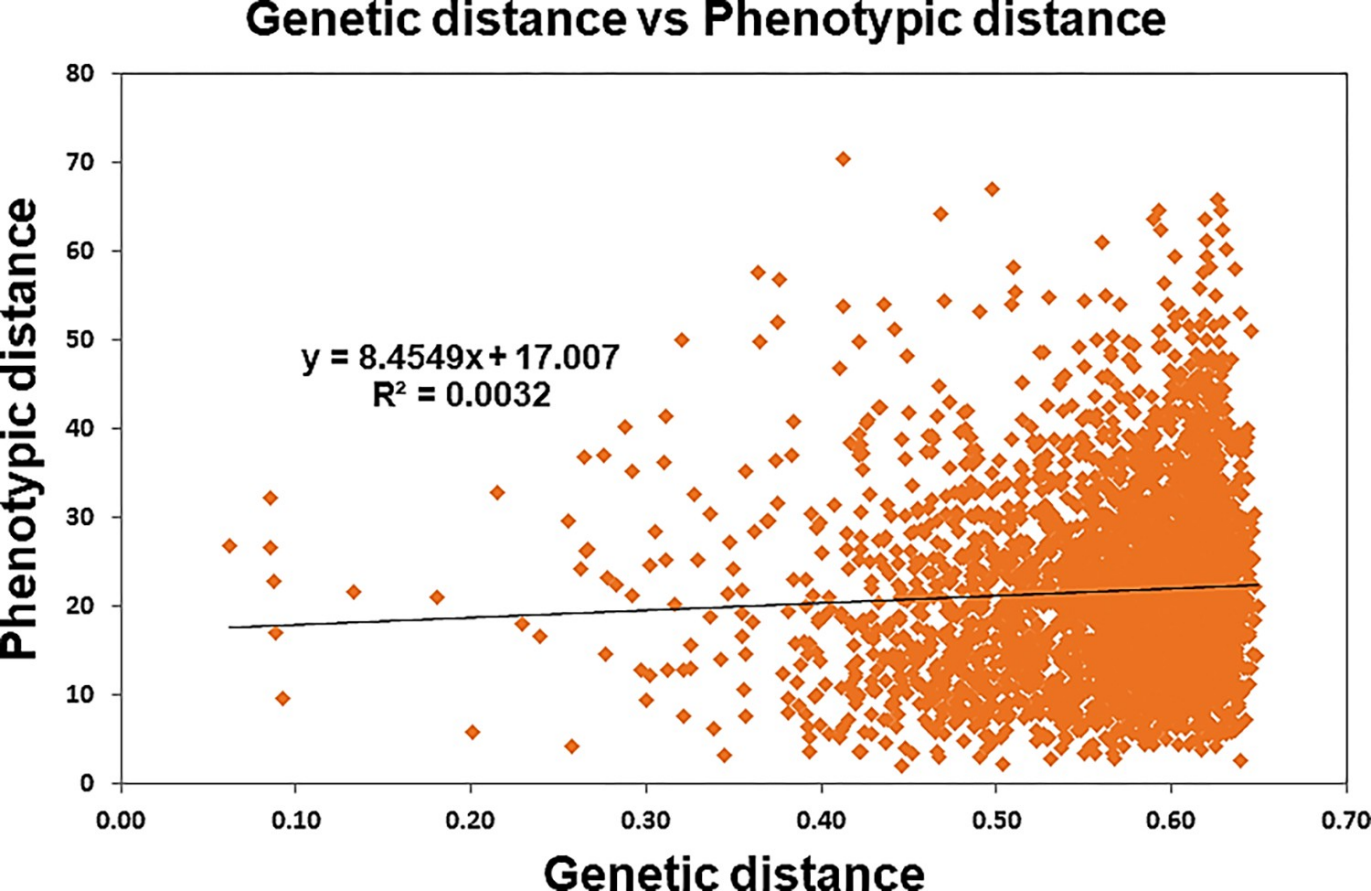
Correlation between SNPs based on Roger’s genetic distance and Mahalanobis D^2^ genetic distance based on phenotypic data using the Mantel test. Every point on the scatter plot signifies a distinct distance data point between two genotypes. The horizontal placement of each point reflects the genetic distance (Roger’s distance), while the vertical position signifies the phenotypic distance (Mahalanobis D^2^ distance).

A significant but weak positive correlation (r = 0.057: P < 0.05) was observed between SNP and morphological traits based on genetic distance (GD) (Fig 9 and S15 Table). This indicated the presence of a weak correlation between the clustering pattern of hybrid parental lines obtained by markers and the morphological traits-based distance matrix. Earlier, [34] reported similar results were reported between SSR-based and phenotype-based genetic distance. One of the probable reasons for this low correlation might be that the loci controlling agronomic traits are under environmental influence, and SNPs are not necessarily associated with this trait and are not influenced by environmental factors [72–75].

**Fig 10:**
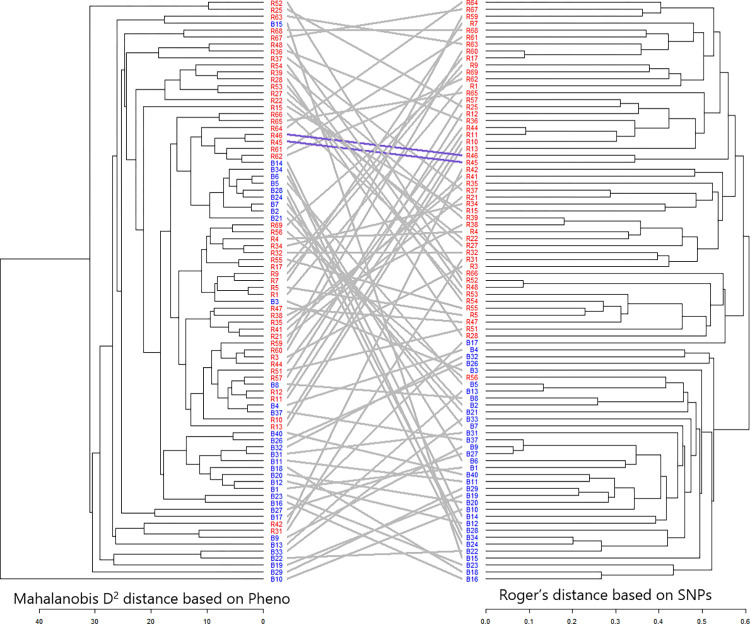
Tanglegram showing the comparison of phenotypic and genotypic dendrograms.

Using the genetic dissimilarity matrix based on combined phenotypic and molecular marker data, the genetic relationship between accessions was analyzed using a comparative approach for hierarchical clustering via a tanglegram (Fig 10 and S15 Table). Using SNP data, B-lines and R-lines were clearly grouped into separate clusters. Although there are almost nine clusters in the case of phenotype-based grouping (Ward’s Method), most of the B-lines and R lines are grouped into separate clusters. Several B-lines, including B14, B34, B6, B5, B28, B24, B7, B2, and B21, were collectively grouped. The likely reason behind this grouping is that these B-lines exhibit characteristics such as late maturity, low dry fodder yield, high HI and shorter plant height. This alignment with the earlier dendrogram, where these lines clustered together in Cluster III, further supports their shared characteristics and suggests a common genetic or phenotypic profile. Tenglegram analysis showed that 36 test accessions maintained their position, which indicated a moderate cophenetic correlation of 43% (36 lines out of 84) between the clustering of hybrid parental lines. A very low positive correlation via the Mantel test and moderate cophenetic correlation in clustering patterns for the B-and R-lines suggests a clear-cut grouping of seed parents and pollinators into separate clusters.

## Conclusion

Knowledge regarding genetic diversity and population structure is of utmost importance in selecting appropriate hybrid parental lines to ensure significant gains from selection and heterosis breeding. The present study investigated the genetic diversity, population structure and genome-wide LD pattern in a 109-pearl millet hybrid parental lines panel having adaptation to drought-prone environments, using 16,472 polymorphic SNP markers. The investigation suggested the existence of two distinct groups, one each for the B- and R-lines. LD analysis showed high to low LD regions across the genome and chromosomes, suggesting a selection history during the breeding process. Diversity based on agro-morphological and grain quality traits showed great variability in existing inbred lines. The genomic and agro-morphological-based characterization delineated lines under investigation into two major sub-populations, one each for seed parents and for pollinators, which agrees with earlier heterotic grouping reports. The two-group strategy can be adopted to develop heterotic pools through well-planned crosses, which can finally be helpful for the better exploitation of heterosis. Greater diversity existed within the population than among the populations, suggesting that these populations could be a valuable source for recombining favourable alleles for hybrid parental development. This study provides valuable information regarding the genetic structure and diversity in parental lines and will help pearl millet breeders to efficiently utilize and select the best parental lines to strengthen breeding pipelines to develop hybrids for drought-prone ecologies.

## Supporting information

S1 Fig Violin plot based on pairwise Nei’ distances of 109 parental lines based on GBS identified 16,472 SNPs; B-lines (41), R-lines (68) and between B and R lines. (DOCX)

S2 Fig The Linkage Disequilibrium pattern based on 16,472 SNPs identified via GBS; is depicted in a Triangle plot for all seven chromosomes. Illustrating LD among SNPs across each chromosome. Pairwise LD values were graphically represented on the X- and Y-axes, with the above diagonal indicating the squared correlation coefficient (r2), and the below diagonal indicating the corresponding P-value.
(DOCX)

S3 Fig Linkage Disequilibrium Decay (LDD) plots, demonstrating a reduction in the squared correlation coefficient (r^2^) between SNP pairs as a function of their physical distance within each chromosome. (DOCX)

S4 Fig Violin plot based on pairwise Modified Rogers’s distances of 84 parental lines based on molecular marker.; B-lines (34), R-lines (50) and between B and R lines. (DOCX)

S5 Fig Violin plot based on pairwise Mahalanobis distances of 84 parental lines based on molecular marker; B-lines (34), R-lines (50) and between B- and R-lines. (DOCX)

S1 Table List of 110 Hybrid parental genotypes along with line code used for Genotyping (GBS) and Field Evaluation during Rainy 2020 and Rainy 2021 under Drought-prone ecology (A1 Zone). (XLSX)

S2 Table Details regarding experimental sites and hybrid parental trials as different sets. (XLSX)

S3 Table Number of single nucleotide polymorphisms (SNPs) detected and distance between SNP markers obtained from genotyping-by-sequencing of 109 genotypes. (XLSX)

S4 Table Chromosome-wise marker frequency for 109 Hybrid parental lines based on 16472 SNP markers. MaF = Major allele frequency, MAF = Minor allele frequency, He = Heterozygosity.
(XLSX)

S5 Table Summary of 16, 472 SNP markers used genotyping 109 hybrid parental lines. (XLSX)

S6 Table Identity-by-State (IBS) based pairwise genetic distance matrix for 109 parental inbred lines obtained from 16,472 SNP markers. (XLSX)

S7 Table Inferred ancestry of individuals and degree of admixture among 109 hybrid parental lines. (XLSX)

S8 Table Nei’s genetic distance matrix for 109 parental inbred lines obtained from 16,472 SNPs. (XLSX)

S9 Table Pedigree of hybrid parental lines in different cluster based on clustering pattern observed in NJ tees based on GBS identified SNPs. (XLSX)

S10 Table Highly genetically dissimilar 25 pairs of hybrid parental lines for use in development of new parental lines and hybrid combination. (XLSX)

S11 Table Percentage of variation explained by the first 3 axes from Principal coordinates analysis (PCoA) for 109 genotypes based on SNP markers. % = Percentage of variation explained by each axis, Cum % = Cumulative Percentage of variation explained by axes.
(XLSX)

S12 Table Combined analysis of variance (ANOVA) and estimates of variance components for Grain yield and related traits for pooled analysis of different sets of parental trials across environments. * p<0.05, ** p<0.01, *** p<0.001; GY = Grain Yield (kg/ha), DFY = Dry Fodder Yiled (Kg/ha), DB = Days to 50% flowering (days), PNHI = panicle harvest Index (%), PH = plant height (cm), TGW = thousand grain weight (gm), Fe = Grain iron content (ppm), Zn = grain zinc content (ppm), PY = panicle yield (kg/ha), HI = Grain harvest Index (%), DTM = Days to maturity (days).
(XLSX)

S13 Table Adjusted means values for 11 phenotypic traits for 87 hybrid parental genotypes. (XLSX)

S14 Table Cluster’s mean based on phenotyping data for yield, grain quality and yield component traits in 84 hybrid parental genotypes. (XLSX)

S15 Table Roger’s Genetic distance (Below diagonal) based on SNP data and Mahalonbis Distance (above diagonal) based on phenotypic data for 84 Hybrid parental lines. (XLSX)
