# Supplementary material for: Understanding genetic diversity in drought-adaptive hybrid parental lines in pearl millet
Source: PLoS One. 2024 Feb 23;19(2):e0298636. doi: 10.1371/journal.pone.0298636 (PMC10890771; doi:10.1371/journal.pone.0298636)
Supplement: S1 Fig — (DOCX) [file pone.0298636.s001.docx]

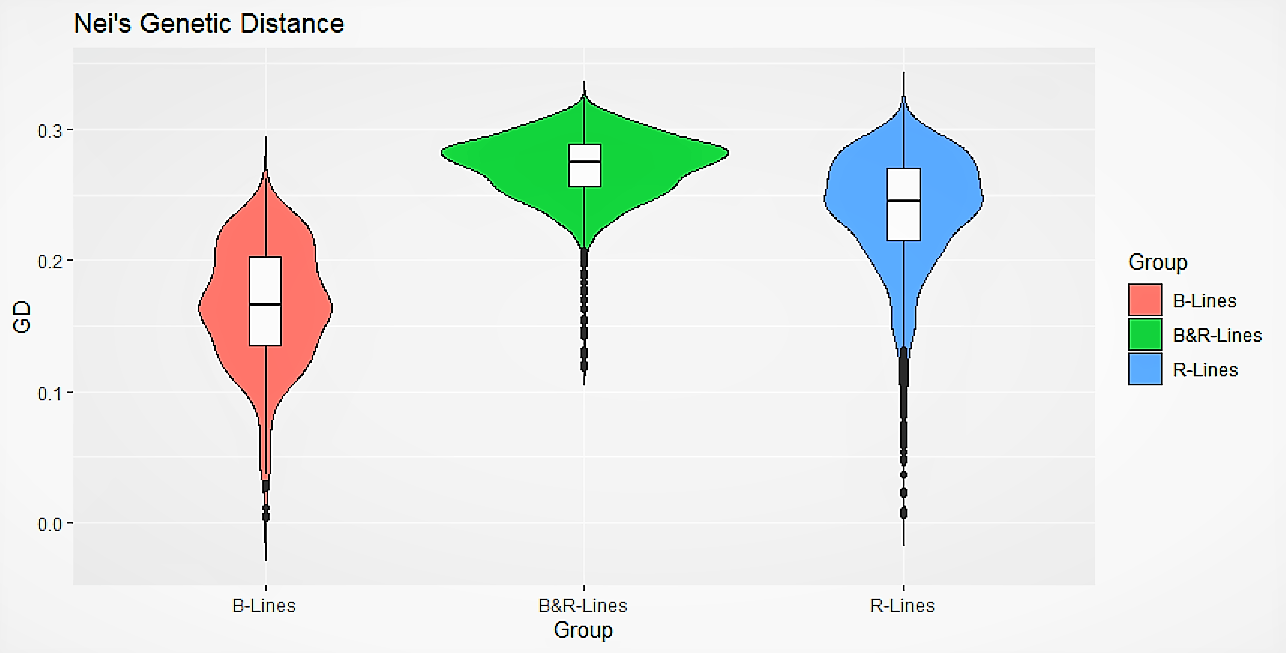
**#PONE-D-23-22980-Supplementary_images**

**S1 Fig.** Violin plot based on pairwise Nei’ distances of 109 parental lines based on GBS identified 16,472 SNPs; B-lines (41), R-lines (68) and between B and R lines.
