# Supplementary material for: Understanding genetic diversity in drought-adaptive hybrid parental lines in pearl millet
Source: PLoS One. 2024 Feb 23;19(2):e0298636. doi: 10.1371/journal.pone.0298636 (PMC10890771; doi:10.1371/journal.pone.0298636)
Supplement: S2 Fig — Illustrating LD among SNPs across each chromosome. Pairwise LD values were graphically represented on the X- and Y-axes, with the above diagonal indicating the squared correlation coefficient (r2), and the below diagonal indicating the corresponding P-value. (DOCX) [file pone.0298636.s002.docx]

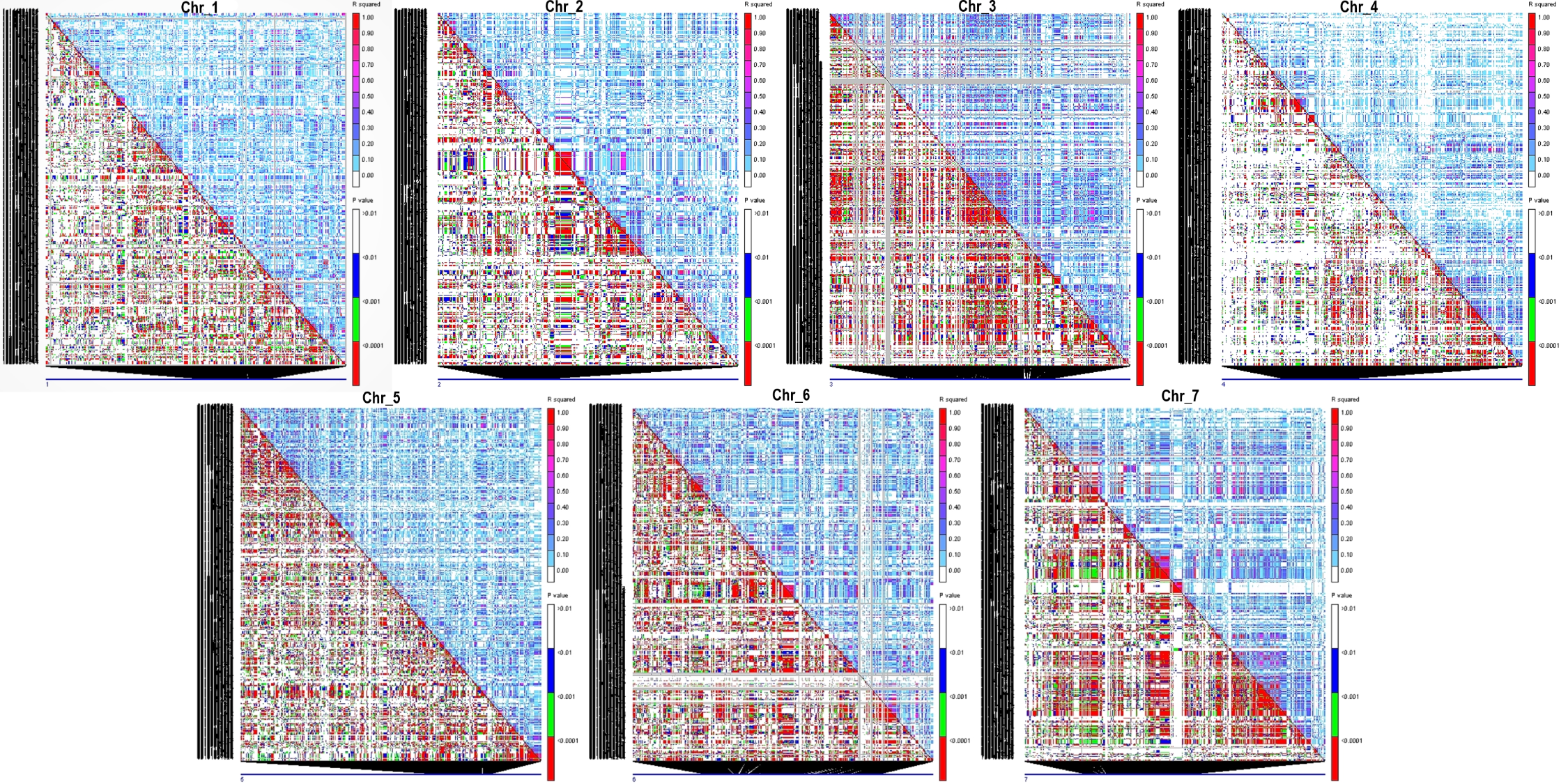
**S2 Fig.** The Linkage Disequilibrium pattern based on 16,472 SNPs identified via GBS; is depicted in a Triangle plot for all seven chromosomes. Illustrating LD among SNPs across each chromosome. Pairwise LD values were graphically represented on the X- and Y-axes, with the above diagonal indicating the squared correlation coefficient (r2), and the below diagonal indicating the corresponding P-value.
