# Supplementary material for: Understanding genetic diversity in drought-adaptive hybrid parental lines in pearl millet
Source: PLoS One. 2024 Feb 23;19(2):e0298636. doi: 10.1371/journal.pone.0298636 (PMC10890771; doi:10.1371/journal.pone.0298636)

**S3 Fig.** Linkage Disequilibrium Decay (LDD) plots, demonstrating a reduction in the squared correlation coefficient (r2) between SNP pairs as a function of their physical distance within each chromosome.
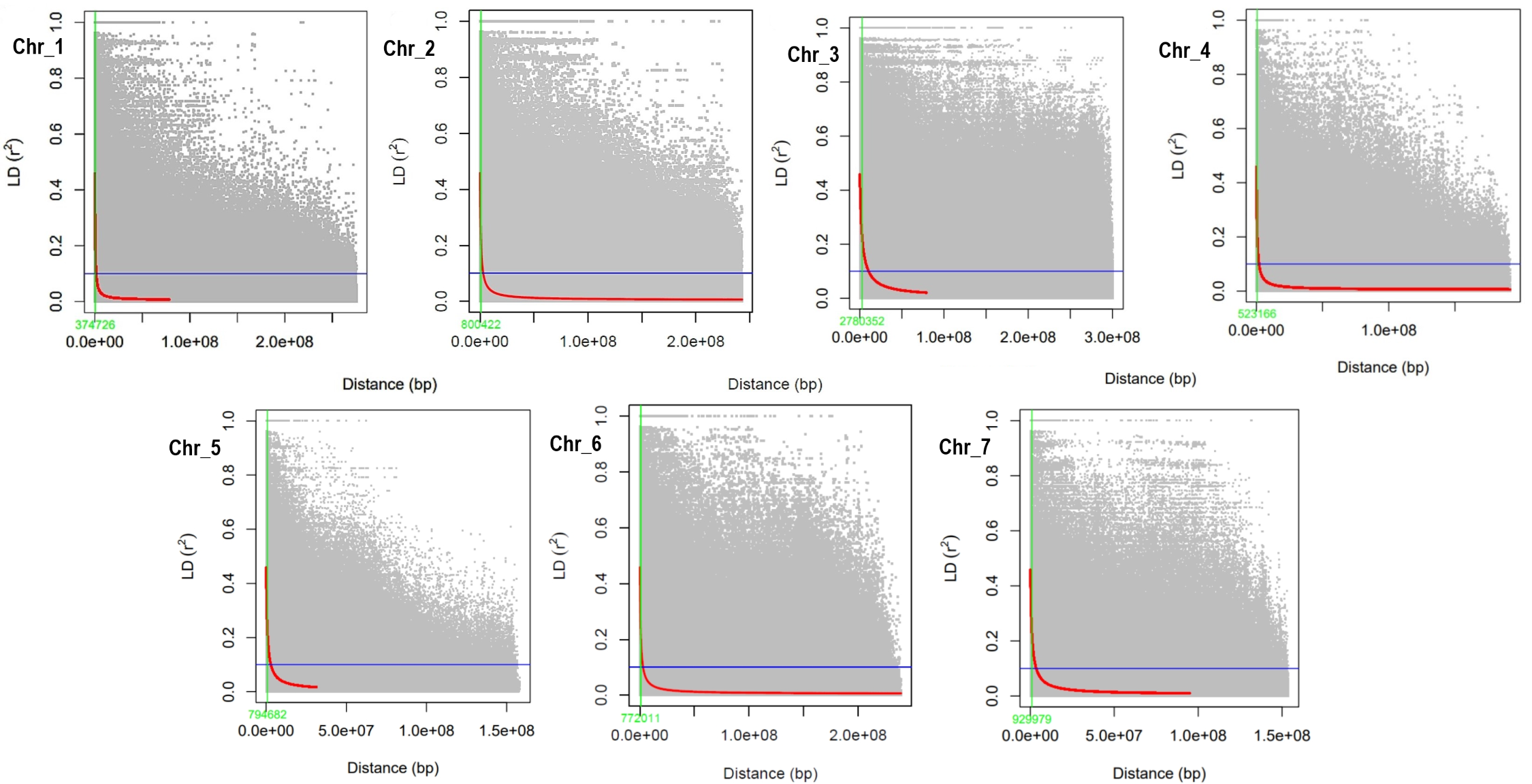

Supplement: S3 Fig — (DOCX) [file pone.0298636.s003.docx]
