# Supplementary material for: Understanding genetic diversity in drought-adaptive hybrid parental lines in pearl millet
Source: PLoS One. 2024 Feb 23;19(2):e0298636. doi: 10.1371/journal.pone.0298636 (PMC10890771; doi:10.1371/journal.pone.0298636)
Supplement: S4 Fig — (DOCX) [file pone.0298636.s004.docx]

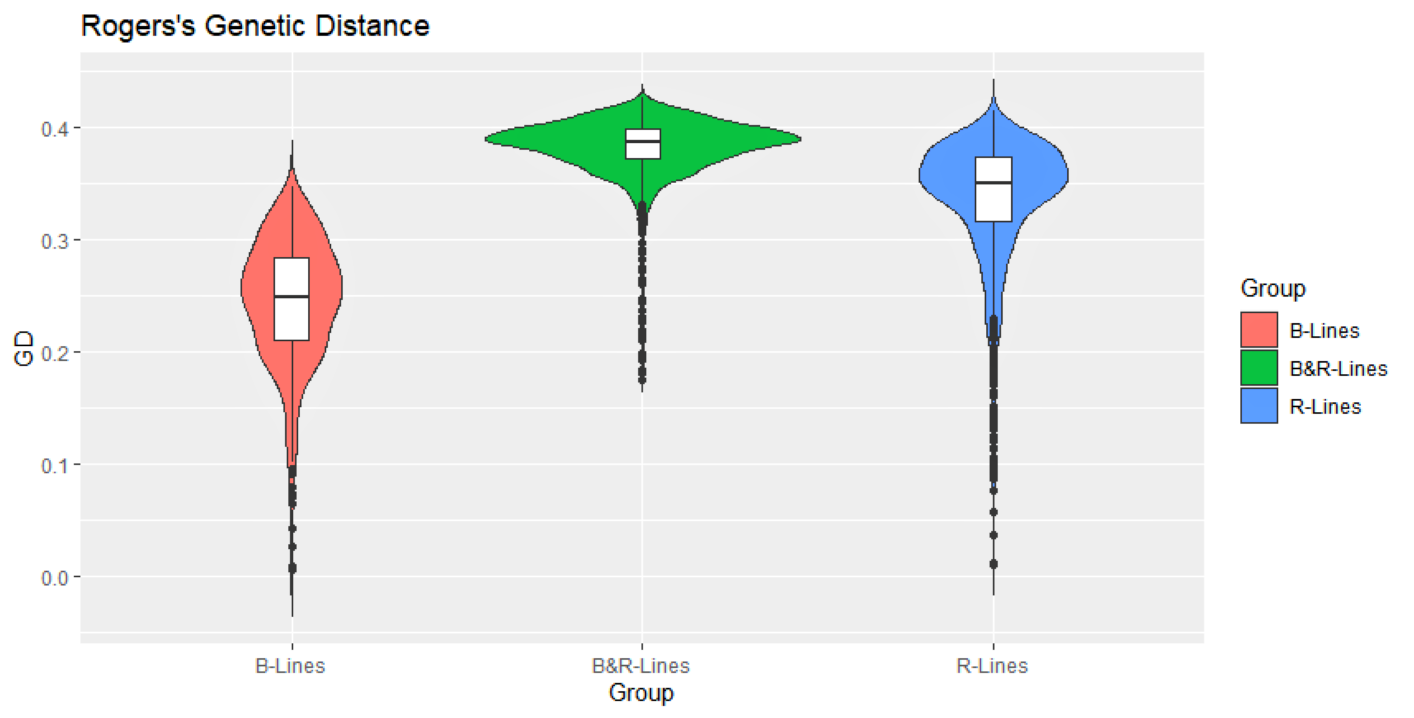
**S4 Fig**. Violin plot based on pairwise Modified Rogers’s distances of 84 parental lines based on molecular marker.; B-lines (34), R-lines (50) and between B and R lines.
