# Supplementary material for: Understanding genetic diversity in drought-adaptive hybrid parental lines in pearl millet
Source: PLoS One. 2024 Feb 23;19(2):e0298636. doi: 10.1371/journal.pone.0298636 (PMC10890771; doi:10.1371/journal.pone.0298636)
Supplement: S5 Fig — (DOCX) [file pone.0298636.s005.docx]

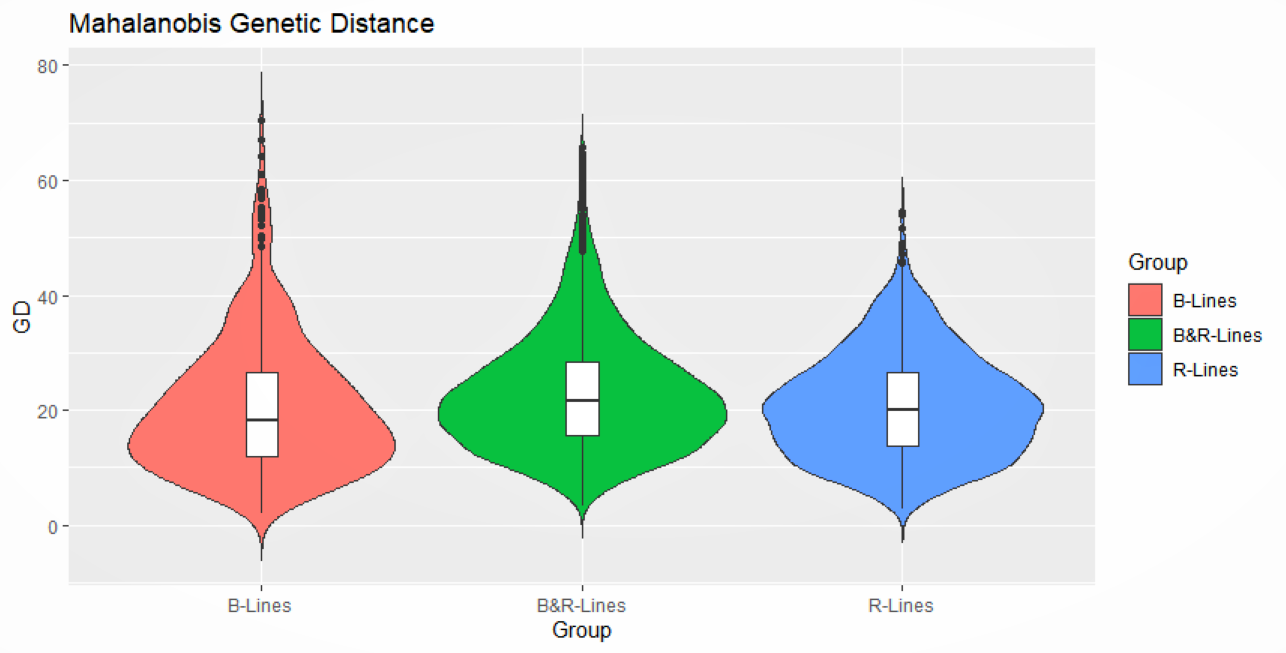


**S5 Fig.** Violin plot based on pairwise Mahalanobis distances of 84 parental lines based on molecular marker; B-lines (34), R-lines (50) and between B- and R-lines
